# Dissecting the Genetic Contribution of Tooth Agenesis

**DOI:** 10.3390/ijms262110485

**Published:** 2025-10-28

**Authors:** Antonio Fallea, Mirella Vinci, Simona L’Episcopo, Massimiliano Bartolone, Antonino Musumeci, Alda Ragalmuto, Simone Treccarichi, Francesco Calì

**Affiliations:** 1 Oasi Research Institute-IRCCS, 94018 Troina, Italy; afallea@oasi.en.it (A.F.); mvinci@oasi.en.it (M.V.); slepiscopo@oasi.en.it (S.L.); mbartolone@oasi.en.it (M.B.); amusumeci@oasi.en.it (A.M.); aragalmuto@oasi.en.it (A.R.); francesco.cali@unikore.it (F.C.); 2 Department of Medicine and Surgery, Kore University of Enna, 94100 Enna, Italy

**Keywords:** dental agenesis, oligodontia, hypodontia, anodontia, next generation sequencing, odontogenesis

## Abstract

Tooth agenesis (TA), the congenital absence of one or more teeth, is the most common manifestation of defective dental morphogenesis in humans. TA can occur as an isolated (non-syndromic) condition or as part of a broader syndromic presentation. In this review, we analyzed a total of 73 manuscripts to provide a comprehensive update on the genetic landscape of TA. To investigate the genes, variants, and associated phenotypes, we reviewed data from curated databases including Human Phenotype Ontology (HPO), OMIM, ClinVar and MalaCards. Based on the current evidence, the genes most frequently implicated in TA are *MSX1*, *EDA*, and *PAX9*. However, chromosomal abnormalities, such as those seen in Down syndrome and Williams syndrome, along with structural variations (e.g., deletions and duplications), also contribute significantly to TA etiology. The most involved pathways include TNF receptor binding, encompassing genes such as *EDA*, *EDA2R*, *EDAR*, and *EDARADD*, and the mTOR signaling pathway, which includes *AXIN2*, *FGFR1*, *LRP6*, *WNT10A*, and *WNT10B*. The aim of this review is to provide an critical synthesis of the genetic mechanisms underlying TA, highlighting the contribution of major signaling pathways, regulatory networks, and emerging molecular insights that may inform diagnostic and therapeutic advances.

## 1. Introduction

Like all developmental processes, odontogenesis is highly complex and tightly regulated, involving the coordinated expression of hundreds of genes in reciprocal signaling networks. Disruptions in these mechanisms—caused by either genetic mutations or environmental factors—can lead to defects in dental development and contribute to common craniofacial anomalies [1,2]. Tooth agenesis (TA), the congenital absence of teeth, is the most common manifestation of defective dental morphogenesis in humans [3]. Particularly, it corresponds to a deficit in the development of a variable number of teeth. TA may affect either the primary or permanent dentition and ranges in severity from hypodontia (five or fewer missing teeth) to oligodontia (six or more missing teeth) and anodontia (complete absence of teeth). TA can present either as a syndromic or non-syndromic condition [4]. Specifically, in the syndromic form, TA occurs as one component of a broader genetic syndrome, often accompanied by craniofacial or systemic abnormalities [3], reflecting specific genotype–phenotype correlations. In contrast, non-syndromic TA appears as an isolated dental anomaly, without other systemic involvement. It is frequently familial and is associated with mutations in genes involved in odontogenesis.

The diagnosis of TA typically requires confirmation through radiographic imaging, such as panoramic X-rays, to accurately assess the number, location, and developmental stage of both erupted and unerupted teeth [2,5].

TA exhibits substantial phenotypic heterogeneity in both the number and distribution of missing teeth. A large cross-sectional study of 861 Caucasian individuals reported a prevalence of 2.78%, with the maxillary arch most frequently affected and bilateral agenesis (83.3%) more common than unilateral forms [6]. The majority of patients (75%) presented with one or two missing teeth—most often the maxillary lateral incisors—indicating a consistent regional vulnerability. While approximately 37.5% of cases followed a familial autosomal dominant inheritance pattern, the remaining were sporadic, suggesting a role for de novo mutations or environmental influences. Importantly, third molar agenesis shows a strong association with other types of TA, as evidenced by its markedly higher prevalence among affected individuals (50.8%) compared to controls (20.5%), reinforcing the hypothesis of shared genetic and developmental determinants underlying these anomalies [7]. Furthermore, in a comprehensive and well-detailed multivariate analysis conducted on 259 individuals (178 controls, 51 with wisdom tooth agenesis, and 38 with agenesis of other teeth), it was identified a robust association between dental agenesis and facial morphology [2]. The findings suggest that transverse constriction of the maxilla, facial divergence, and anterior projection of the chin symphysis are associated with dental agenesis.

Studies have reported significant gender differences in patterns of non-syndromic TA. A retrospective study found that females had a higher overall prevalence of TA, particularly in cases involving multiple missing teeth, whereas single-tooth agenesis was more common in males [5]. A cross-sectional study further confirmed this sexual dimorphism, noting specific patterns of lateral incisor agenesis: the maxillary left lateral incisor was more frequently missing in males (*p* = 0.019), while the mandibular right lateral incisor and bilateral agenesis of mandibular lateral incisors were more common in females (*p* = 0.025 and *p* = 0.015, respectively) [8]. Notably, unilateral agenesis of the mandibular left lateral incisor was observed exclusively in males (*p* = 0.047). These findings support the potential presence of gender-specific patterns in TA. Nevertheless, we emphasize that larger and more diverse cohorts are needed to validate these associations.

Genome-wide association studies (GWAS) have emerged as a key approach for elucidating the genetic architecture of TA by the identification susceptibility loci across large patient cohorts. Recent analyses have uncovered multiple SNPs and genes significantly associated with TA, underscoring the complex polygenic nature of this condition. In a large Chinese cohort, four SNPs on chromosome 2—including rs147680216 within *WNT10A*—were strongly linked to premolar agenesis, reinforcing the central role of Wnt pathway dysregulation in tooth development [9]. Additional loci, such as rs6738629 near GCC2, were also implicated in craniofacial morphogenesis. Similarly, associations involving *ADAMTS9*, *PRICKLE2*, *BMP7*, *MSX2*, and *ROBO1*/*2* suggest overlapping genetic mechanisms between TA and other dental anomalies [10]. Complementary GWAS data identified further variants, including rs4498834 (*ASCL5*/*CACNA1S*), rs35822372 (*FOXI3*), chr2:108,896,996 (*EDAR*), and rs2034604 (*ARHGAP15*), which highlight the contribution of ectodysplasin signaling and cytoskeletal organization to odontogenesis. Moreover, recurrent WNT10A variants (rs121908120 and rs121908119) and rs371555610 in *ZFHX4* further support the involvement of Wnt signaling and craniofacial transcriptional regulation in TA pathogenesis [11]. Collectively, these findings confirm that TA arises from the interplay of multiple signaling pathways—including Wnt and *EDA*/*EDAR*—and emphasize its genetically heterogeneous nature.

As extensively outlined, congenital TA may have a multifactorial etiology which encompasses genetic, epigenetic, and environmental influences [1,3,12]. Notably, the genetic landscape of TA can be explored through several advanced genomic approaches, such as whole-exome sequencing (WES), whole-genome sequencing (WGS), and GWAS. Through these methods, multiple genes have been identified as strong candidates for TA, including *MSX1*, *PAX9*, *AXIN2*, *PITX2*, *WNT10A*, *NEMO*, *EDA*, *EDAR*, *EDARADD*, *GREMLIN2*, *LTBP3*, *LRP6*, and *SMOC2* [13].

This review aims to provide an updated and critical synthesis of the genetic mechanisms underlying TA, highlighting the contribution of major signaling pathways, regulatory networks, and emerging molecular insights that may inform diagnostic and therapeutic advances.

### Search Strategy

A total of 73 manuscripts met the inclusion criteria, including narrative and systematic reviews, as well as original research articles encompassing observational and cross-sectional studies published between 2016 and 2025 (date of last search 6 October 2025). Literature searches were performed in PubMed and Google Scholar using keyword combinations and Boolean operators (“AND”, “OR”), with terms such as: ‘tooth agenesis’, ‘genetic dental agenesis’, ‘dental agenesis’, ‘Down syndrome dental agenesis’, ‘artificial intelligence in dentistry’, ‘tooth eruption’, ‘GWAS AND tooth agenesis’, ‘Wolf-Hirschhorn AND tooth agenesis’, ‘small RNA AND tooth agenesis’, and ‘GWAS AND tooth agenesis’. Manuscripts were subsequently screened and selected manually. Although no language filters were applied during the search process, only articles published in English were included. In addition, the database MalaCards (https://www.malacards.org/) (Version 5.25, released 23 July 2025) (accessed on 1 September 2025) was searched using the term “tooth agenesis,” identifying 141 inferred genes and the top 10 pathways most strongly associated with the condition. The Human Phenotype Ontology (HPO) database (https://hpo.jax.org/) (Version 2.0.6) (accessed on 1 September 2025) was queried using the term “tooth agenesis”, yielding 351 genes and 361 diseases associated with this condition. The analysis further explored the HPO hierarchy related to abnormality of number of teeth (HP:0006483) and abnormality of dental eruption (HP:0006292), from which the following categories were selected for detailed evaluation: tooth agenesis (HP:0001592), impacted tooth (HP:0011079), agenesis of maxillary incisor (HP:0200160), and eruption failure (HP:0000706). OMIM database (https://www.omim.org/) (accessed on 1 September 2025) was queried for the entries corresponding to the diseases associated with the TA categories identified from the HPO database.

## 2. Phenotypic Variability

### 2.1. Different Types of Dental Agenesis

As previously noted in the Introduction, three main types of dental agenesis can be distinguished based on severity. TA may affect either the primary or permanent dentition and is classified as

- Hypodontia: the absence of five or fewer teeth,
- Oligodontia: the absence of six or more teeth
- Anodontia: the complete absence of all teeth.

TA presents a wide array of associated phenotypes, reflecting its underlying genetic complexity and variable expressivity. The most affected teeth are the second premolars and maxillary lateral incisors, though the pattern and severity of tooth loss can range from the absence of a single tooth (hypodontia) to complete anodontia [14]. TA is frequently associated with other dental anomalies, such as microdontia, taurodontism, delayed eruption, transposition, and altered tooth morphology [14,15]. Craniofacial malformations are frequently associated with TA, particularly in syndromic cases [16]. Structural anomalies such as midfacial hypoplasia, and mandibular dysmorphisms often coexist with missing teeth, reflecting shared developmental pathways during craniofacial and dental morphogenesis. These craniofacial malformations can disrupt normal eruption patterns, alter occlusion, and contribute to functional issues such as speech difficulties and impaired mastication. As documented in the literature, although the exact etiology remains unclear, TA and maxillary growth restriction are well-recognized sequelae in patients with unilateral cleft lip and/or palate [17,18]. Intrinsic factors appear to contribute significantly, as the severity of maxillary hypoplasia tends to increase with the extent of dental agenesis [17,18]. In a clinical observational study involving 88 individuals with CLP, agenesis of lateral incisors was observed in approximately 69% of unilateral CLP cases, 78% of bilateral CLP cases, and 18% of patients with isolated cleft palate [17].

The coexistence of TA with craniofacial anomalies underscores the importance of a multidisciplinary diagnostic approach integrating dental, genetic, and craniofacial assessments.

### 2.2. Comorbidity with Other Diseases

Tooth agenesis is associated with a wide range of diseases and syndromes. According to the Human Phenotype Ontology (HPO) database, TA is observed across multiple phenotypic categories, including selective tooth agenesis (HP:0001592), impacted tooth (HP:0011079), agenesis of maxillary incisor (HP:0200160), and eruption failure (HP:0000706). This broad phenotypic distribution has also been confirmed by a recent systematic review [19]. However, despite some overlapping in clinical features, not all phenotypes involving TA are classified under the specific term “tooth agenesis.” Table 1 summarizes all HPO-annotated conditions involving TA, along with the number of associated diseases and related genes.

**Figure 1:**
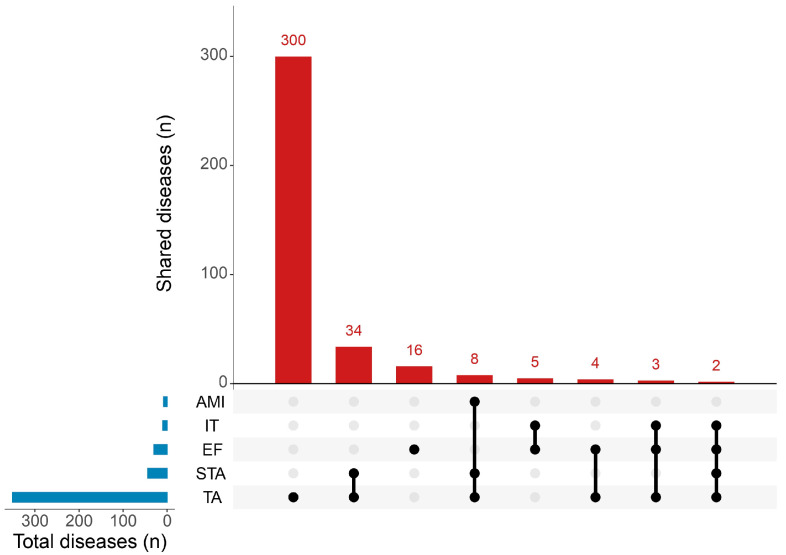
Graphical representation of the number of associated diseases shared across tooth agenesis, selective tooth agenesis, impacted tooth, agenesis of maxillary incisor and eruption failure. Data were retrieved from HPO database (https://hpo.jax.org/) and subsequently elaborated using R studio version 3.4.3.

All the diseases and genes associated with the listed phenotypes are reported in Supplementary File (Tables S1 and S2). On the other hand, Figure 1 shows the shared diseases across these 5 conditions, emphasizing the variable number of phenotypes associated with TA.

Elsahy–Waters syndrome (OMIM: 211380) and Otopalatodigital syndrome type I (OPD1) (OMIM: 311300) are the two syndromes most strongly associated with multiple TA phenotypes, including tooth agenesis, selective tooth agenesis, impacted teeth, and eruption failure. This syndrome is a rare autosomal recessive disorder caused by loss-of-function variants in the *CDH11* gene, which encodes cadherin 11. Clinically, affected individuals present with brachycephaly, midface hypoplasia, distinctive craniofacial features, cervical vertebral fusion, 2–3 digit syndactyly, genitourinary anomalies, and intellectual disability. Notably, dental anomalies—particularly tooth agenesis and tooth loss—are consistent features of the phenotype [20,21]. According to OMIM database, Otopalatodigital syndrome, type I is an X-linked dominant (XLD) associated with mutations in *FLNA* gene. Males with OPD1 have cleft palate and mild skeletal anomalies with conductive deafness caused by ossicular anomalies and cleft palate. This syndrome often presents with selective tooth agenesis.

**Figure 2:**
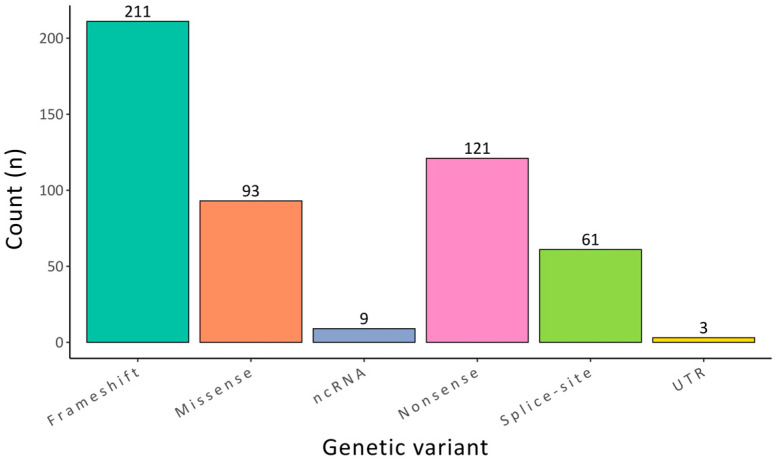
Graphical representation of the distribution of the 401 germline genetic variants classified as likely pathogenic or pathogenic in the ClinVar database for tooth agenesis (TA). The most frequent variant types include frameshift and missense mutations, highlighting the genetic heterogeneity underlying TA.

As evidenced in Figure 2, eight rare disorders share the overlapping phenotypes of tooth agenesis, selective tooth agenesis, and agenesis of the maxillary incisor, suggesting a convergence of pathogenic mechanisms affecting odontogenesis. Among these:

- Trichothiodystrophy 2, photosensitive (OMIM: 616390) is an autosomal recessive disorder caused by mutations in *ERCC3*/*XPB* (2q14), which encodes a helicase subunit of the transcription/repair factor TFIIH. Patients exhibit brittle, sulfur-deficient hair with the characteristic “tiger-tail banding” pattern under polarized light, along with agenesis of the second upper incisor. Additional features include ichthyosis, intellectual or developmental disabilities, decreased fertility, ocular abnormalities, short stature, and recurrent infections.
- Arthrogryposis, distal type 12 (OMIM: 620545) is caused by homozygous mutations in ADAMTS15 (11q25) and is associated with craniofacial and skeletal anomalies together with tooth agenesis, particularly involving the maxillary incisors.
- Pallister-W syndrome (ORPHA: 2804) is a rare congenital disorder characterized by moderate to severe intellectual disability, seizures, spasticity, strabismus, and facial dysmorphism. Although the underlying genetic defect remains unknown, an X-linked inheritance pattern has been suggested. Dental anomalies, including maxillary incisor agenesis, are frequently described.
- Peters-plus syndrome (OMIM: 261540) results from homozygous or compound heterozygous mutations in *B3GALTL* (13q12). It is characterized by ocular malformations, cleft lip/palate, short stature, brachydactyly, and developmental delay. Tooth agenesis has also been consistently reported.
- Bloom syndrome (OMIM: 210900), caused by homozygous or compound heterozygous mutations in *RECQL3* (15q26), is an autosomal recessive condition marked by growth retardation, photosensitive skin changes, immunodeficiency, insulin resistance, and a high risk of multiple early-onset cancers due to chromosomal instability. Hypodontia, particularly involving the upper lateral incisors, is a common finding.
- Microphthalmia, syndromic 1 (OMIM: 309800) is an X-linked disorder caused by mutations in *NAA10* (Xq28). It presents with unilateral or bilateral microphthalmia or anophthalmia, together with developmental delay, dysplastic ears with skin tags, cleft palate, urogenital anomalies, skeletal defects, and dental anomalies including agenesis.
- CHAND syndrome (OMIM: 214350; ORPHA: 1401) is an autosomal recessive disorder caused by homozygous mutations in *RIPK4* (21q22). It is characterized by ankyloblepharon, sparse curly hair, nail dysplasia, oral frenula, and dental agenesis.

On the other hand, three disorders share the overlapping phenotypes of tooth agenesis, impacted tooth, and eruption failure, highlighting their impact on dental development and craniofacial growth. These include the following:

- Cherubism (OMIM: 118400) is caused by heterozygous mutations in SH3BP2 (4p16). The condition is characterized by progressive bone loss confined to the jaws, replaced by fibrous tissue, resulting in bilateral facial swelling. According to the OMIM clinical synopsis, affected individuals may present with oligodontia, tooth agenesis, and displaced or impacted teeth.
- Eiken syndrome (OMIM: 600002) is a rare autosomal recessive skeletal dysplasia caused by homozygous mutations in PTHR1 (3p21). It is defined by delayed bone ossification, epiphyseal dysplasia, and abnormalities in bone remodeling, with dental phenotypes that include agenesis and eruption defects.
- Hutchinson–Gilford progeria syndrome (HGPS) (ORPHA: 740; OMIM: 176670) arises from de novo heterozygous mutations in LMNA (1q22). While Orphanet describes both autosomal dominant and recessive inheritance patterns, HGPS is typically sporadic. Clinically, it is characterized by short stature, alopecia, lipodystrophy, scleroderma-like skin, joint stiffness, osteolysis, and a prematurely aged facial appearance. As reported in OMIM, dental findings include delayed eruption, hypodontia, and crowding.

As previously noted in the Introduction, craniofacial deformities—particularly those observed in microcephaly, such as reduced anterior facial height, a smaller gonial angle, and a decreased mandibular plane angle, have been significantly associated with dental abnormalities, including delayed eruption of primary teeth and TA [22,23]. Within this context, microcephaly was defined as a risk factor for dental alterations [23].

Intellectual disability (ID), is often associated with TA. Precisely, only few studies have explicitly investigated the association between ID and TA. It is important to clarify that TA may have a monogenic, polygenic, or chromosomal etiology, with Down syndrome (DS) being a common chromosomal cause. While dental problems such as dental caries, tooth loss, and interproximal bone loss are frequently reported in individuals with ID [24,25], an explicit association between ID and TA has primarily been observed in individuals with DS or other chromosomal abnormalities. It is noteworthy that other syndromes characterized by ID of varying severity—often accompanied by facial dysmorphisms—may also include TA among their features. For example, Kabuki syndrome (MIM #147920), which presents with ID, hypotonia, and distinctive craniofacial and skeletal features, has been associated with TA, although with variable phenotypic expression [26]. As described later in this review, Kabuki syndrome is caused by mutations in the *KMT2D* gene. In a retrospective study analyzing oral features in eight patients with Kabuki syndrome (KS), the most common findings included dental caries (n = 5) and tooth agenesis (n = 5) [27]. Other anomalies observed were delayed tooth eruption (n = 4), cleft lip/palate (n = 2), enamel hypoplasia (n = 2), tooth fusion (n = 1), and microdontia (n = 1).

### 2.3. Chromosomal Disorders Associated with Dental Agenesis

The most extensively studied chromosomal anomaly linked to permanent TA is Down syndrome (DS). DS, caused by trisomy of chromosome 21, is the most common chromosomal disorder in newborns and impacts both physical and intellectual development [28]. Radiographic studies in individuals with DS have shown that midfacial underdevelopment can lead to malocclusion, delayed eruption of permanent teeth, a high prevalence of hypodontia and microdontia, altered crown morphology, thinner dentin and enamel, taurodontism, tooth transpositions, and bruxism [29]. These anomalies can significantly compromise the quality of life of individuals with DS by impacting eating and swallowing processes. A meta-analysis estimated the overall prevalence of permanent tooth agenesis (excluding third molars) in individuals with DS to be 54.6%, although considerable heterogeneity was observed [30]. Among affected individuals, approximately half presented with three or more missing teeth. The permanent teeth most frequently absent were the maxillary lateral incisor (27%), mandibular second premolar (21%), and maxillary second premolar (18%) [30]. Notably, the single most commonly missing tooth was the mandibular left second premolar (19.9%), followed closely by the maxillary left lateral incisor (19.4%) [30]. Another scoping review supports the previously described trend of a high prevalence of dental anomalies in individuals with DS, reporting rates ranging from 50.47% to 95.52% [15]. Within this broad range, a complex heterogeneity of anomalies was observed. Specifically, taurodontism was present in 9.52–85.71% of cases, anodontia in 34.69%, delayed eruption in 2.04%, conical teeth in 14.28%, microdontia in 2.04–16.19%, tooth fusion in 2.04%, hypodontia in 16.19–62%, fissured tongue in 78%, retained teeth in 10.17%, and tooth agenesis in 30–81% of cases [15].

In a comparative retrospective study involving 1067 individuals, the association between TA and other disorders was statistically analyzed [31]. The prevalence of TA in children with systemic diseases or congenital malformations (SD/CM) was significantly higher at 19.8%, compared with 9.7% in healthy controls (*p*  <  0.05). Within the SD/CM group, children with DA presented with ectodermal dysplasia (4.4%), DS (8.2%), cleft lip and palate (4.4%), intellectual disability/developmental delay (16.4%), and other genetic or organic diseases without intellectual disability (45.9%) [31].

As extensively documented, Wolf–Hirschhorn syndrome (WHS)—a rare chromosomal disorder caused by a deletion on the distal short arm (p) of chromosome 4—is associated with TA. A hallmark feature of WHS is the characteristic “Greek warrior helmet” facies, along with developmental delay and ID. Approximately 50% of affected individuals present with oral anomalies, most commonly delayed tooth eruption, dental agenesis, and micrognathia [1,32,33]. The likely cause of TA in WHS has been attributed to haploinsufficiency of the *MSX1* gene, which plays a critical role in odontogenesis and craniofacial development. Loss of *MSX1* function may also contribute to cleft lip and/or palate, further supporting its central role in dental and facial morphogenesis. *MSX1* will be further discussed in this review, due to its robust correlation with tooth development and TA. As documented in a cross-sectional study involving 31 individuals, the most prevalent oral manifestations were delayed tooth eruption (74.1%), bruxism (64.5%), dental agenesis (63.6%), micrognathia (60.0%), oligodontia (45.5%), and downturned corners of the mouth (32.3%) [32].

Another chromosomal anomaly in which TA may occur is Turner Syndrome (TS)—a rare disorder in females caused by partial or complete monosomy of the X chromosome, frequently associated with oral and craniofacial anomalies. In a cohort study of 15 TS patients, tooth agenesis was identified as a recurrent finding, along with supernumerary molars, microdontia, enamel defects, and altered eruption patterns [34]. Notably, anomalies in tooth number—including agenesis of maxillary incisors and supernumerary molars—were observed in 42.8% of the subjects. Despite the well-established link between TS and various dental abnormalities, reports specifically addressing the association between TS and TA remain limited in the current literature.

Williams syndrome (WS) is a rare genetic disorder caused by a microdeletion at chromosome 7q11.23, affecting 26–28 genes, including *ELN*, which is essential for elastin production [35]. It is characterized by cognitive impairment and congenital heart disease, present in nearly all affected individuals. While malocclusion is the most frequently reported dental feature, other anomalies such as hypodontia (commonly involving incisors), microdontia, and generalized diastemas are also prevalent. In an observational study of 52 individuals with WS, 98% exhibited at least one dental developmental anomaly, with diastemas (72.5%) and hypodontia (50.9%) being the most common [36]. These findings highlight the need for early and comprehensive dental assessment in individuals with WS.

DiGeorge syndrome (DGS), also known as 22q11.2 deletion syndrome, is a chromosomal disorder strongly associated with dental anomalies [37,38,39]. It is primarily a T-cell immunodeficiency disorder with a broad and heterogeneous clinical spectrum, often including craniofacial abnormalities. As reported in a comprehensive review, TA occurs in approximately 20% of individuals with DGS [38]. Additionally, features of the oral phenotype may include delayed eruption, as documented in case reports [40], further supporting the involvement of 22q11.2 deletion in dental development.

Despite its less frequent association with TA, Cri-du-chat syndrome is a rare chromosomal disorder caused by a deletion on the short arm of chromosome 5 (5p). It is characterized by distinctive craniofacial anomalies, including microcephaly, hypertelorism, and a round face, along with severe intellectual disability [41,42]. While dental anomalies such as delayed eruption and malocclusion are common, dental agenesis has been reported less frequently in this population [42,43].

Table 2 summarizes the chromosomal abnormalities discussed in this subsection, highlighting the specific genetic alterations and their associated dental anomalies.

## 3. Genetic Insights of Dental Agenesis

### 3.1. Genes and Polymorphisms Associated with Dental Agenesis

Several studies and meta-analyses have documented a wide array of genes associated with TA, particularly in its more severe forms such as oligodontia. The most consistently reported candidate genes include *MSX1*, *PAX9*, *AXIN2*, *PITX2*, *WNT10A*, and *EDA*, which are involved in key signaling pathways regulating tooth development. Additional genes such as *NEMO*, *EDAR*, *EDARADD*, *GREMLIN2*, *LTBP3*, *LRP6*, and *SMOC2* have also been implicated in various clinical presentations of TA [13,44].

Further, a comprehensive review highlighted the association between gene mutations in conserved signaling pathways (*WNT*, *EDA*, *SHH*, *FGF*, and *TGF*-β/BMP) and key regulatory molecules (*PAX9*, *PITX2*, *IRF6*, the p53 family, and subunits of RNA polymerase III) as primary causes of syndromic TA [1].

According to the ClinVar database, all reported genetic variants associated with TA are of germline origin. Of these, 401 variants are classified as pathogenic and 179 as likely pathogenic. This set includes 211 frameshift, 121 nonsense, 93 missense, 61 splice-site, 9 non-coding RNA (ncRNA), and 3 untranslated region (UTR) variants. These findings underscore the significant genetic heterogeneity of TA and highlight the diverse mutational mechanisms underlying its etiology. Figure 2 graphically shows the distribution of likely pathogenic and pathogenic genetic variants associated with TA.

According to the MalaCards database, 141 genes have been inferred to be associated with TA, reflecting the condition’s significant genetic heterogeneity. The association scores range widely from a high of 901.83 for *MSX1*, a well-established regulator of odontogenesis, to a low of 4.94 for *WNT7A* and *SMAD9*, suggesting varying degrees of evidence supporting their involvement. Notably, *SUMO1* ranks as the 16th most associated gene, with a considerably lower score of 28.81, indicating a moderate connection to the condition. This disparity in association strength highlights the need to distinguish between core pathogenic genes—those directly involved in tooth development pathways—and secondary or modifier genes, which may influence susceptibility or phenotype expression under specific genetic or environmental contexts. For instance, *SUMO1*, a gene more commonly implicated in cleft lip and palate, may contribute to TA indirectly through its role in craniofacial morphogenesis and epithelial–mesenchymal interactions rather than through direct regulation of tooth morphogenesis [45]. Conversely, *WNT7A* and *SMAD9* appear to contribute to tooth development through their roles in dental epithelial differentiation during embryogenesis. Mutations in *WNT7A* are known to cause Al-Awadi–Raas–Rothschild syndrome, a rare congenital limb malformation disorder, in which dental anomalies such as tooth agenesis, taurodontism, and microdontia have also been reported [1,46]. *SMAD9*, a downstream effector of the BMP/TGF-β signaling pathway, has been predicted to regulate a broad set of genes involved in dental epithelial differentiation and morphogenesis, suggesting its potential modulatory role in odontogenesis [47]. This wide distribution of association scores highlights the complex genetic architecture underlying TA, where a few genes show strong direct involvement, while many others may contribute more subtly or contextually. Table 3 lists the top 15 genes associated with dental agenesis in MalaCards database.

*PAX9* is one of the most inferred genes associated with TA. According to the ClinVar database, 43 TA genetic variants have been associated with TA or related phenotypes (oligodontia, hypodontia), including 10 classified as likely pathogenic and 33 as pathogenic. Notably, most of these variants are either frameshift (16) or missense (16), highlighting the critical impact of protein-altering mutations in TA pathogenesis.

According to the ClinVar database, 21 variants of the *EDA* gene are classified as likely pathogenic (5) or pathogenic (18), with 19 of these being missense mutations.

In the ClinVar database, 10 *MSX1* variants have been reported in association with dental agenesis, all classified as pathogenic. Among these, 3 are missense mutations and three are frameshift mutations, underscoring the gene’s critical role in tooth development.

**Figure 3:**
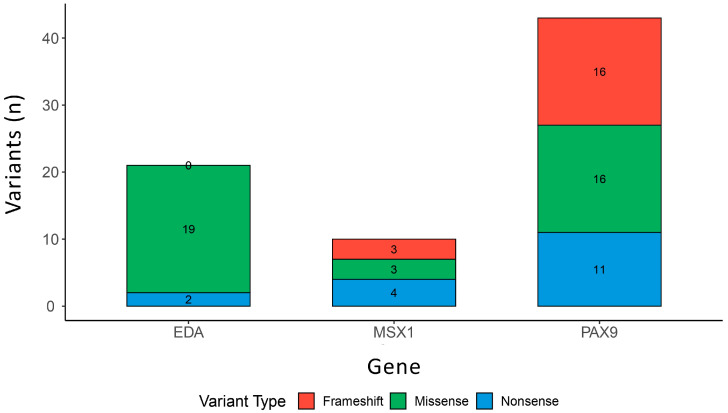
Distribution of likely pathogenic and pathogenic variant types in *PAX9*, *EDA*, and *MSX1* genes. This bar plot shows the number of frameshift, missense, and nonsense variants associated with dental agenesis, as reported in the ClinVar database (https://www.ncbi.nlm.nih.gov/clinvar/) (accessed on 1 September 2025). *PAX9* and *EDA* exhibit a higher proportion of protein-altering mutations, with missense variants being the most frequent in *EDA*. The data highlights the distinct mutational spectra of these key genes involved in tooth development.

Figure 3 visually illustrates the distribution of these variant types across the three genes.

**Figure 4:**
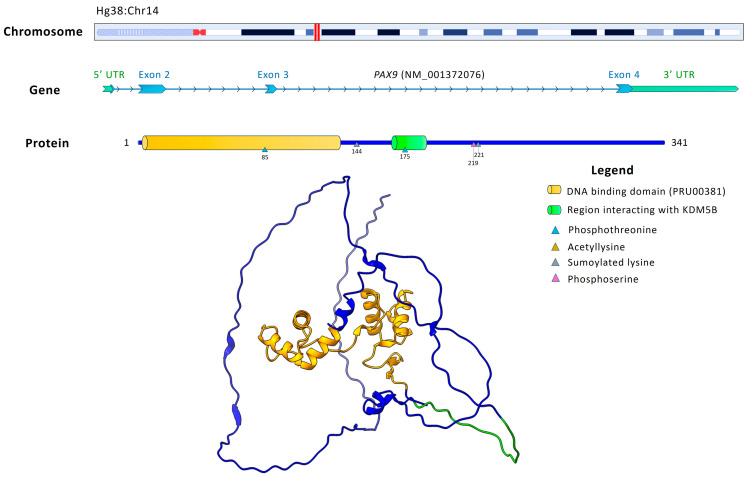
Genomic organization and protein structure of *PAX9*. The top panel shows the chromosomal position (marked by red lines) of *PAX9* in chromosome 14. The gene consists of four exons, represented in light blue. The protein comprises 341 amino acids and includes a paired-type DNA-binding domain (yellow, PRU00381) and a region interacting with KDM5B (green). Post-translational modifications are highlighted: phosphothreonine (light blue triangle), acetyllysine (yellow triangle), sumoylated lysine (gray triangle), and phosphoserine (pink triangle). Extra domain regions are depicted in blue.

According to QuickGO, PAX9 (UniProt P55771) is annotated with transcriptional functions including DNA-binding transcription factor activity, RNA polymerase II-specific (GO:0000981) and cis-regulatory region sequence-specific DNA binding (GO:0001228), underscoring its critical role in odontogenesis and dental morphogenesis. Figure 4 schematizes the genomic organization of *PAX9*, including its chromosomal localization, gene structure, and protein domains.

As previously documented, a meta-analysis of 522 patients examined the number and types of missing teeth in relation to specific gene mutations, highlighting that all identified genes—except *PAX9*—were linked to a broader spectrum of syndromes, while *PAX9* mutations were uniquely associated with isolated TA [13]. As documented in a study carried out using *Pax9*-deficient mouse models [48], *PAX9* plays a crucial role in palate morphogenesis. Its deficiency results in a marked reduction in Axin2 expression, a well-established downstream target of the canonical Wnt signaling pathway, thereby disrupting Wnt activity and impairing normal craniofacial and dental development. Pax9-deficient mice show missing teeth, specifically lacking molars, matching the human pattern [49].

**Figure 5:**
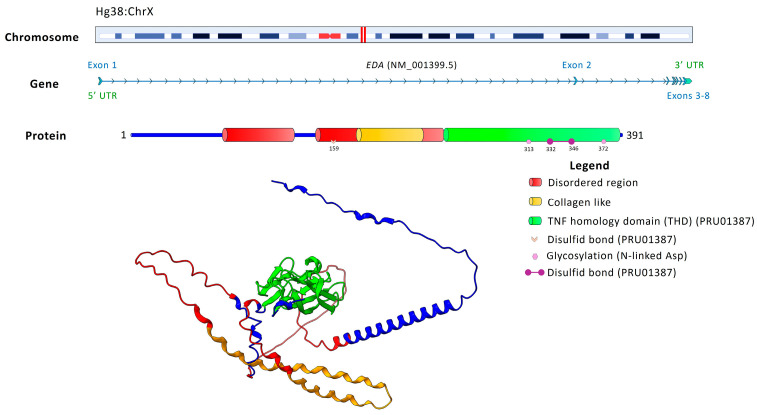
Chromosomal localization, gene structure, and protein domains of *EDA* (NM_001399.5). The upper panel shows the localization of the *EDA* gene on chromosome X, with its position highlighted in red. The gene consists of 8 exons. The encoded protein is composed of 391 amino acids and contains key functional regions, including disordered regions (red), a collagen-like domain (yellow), and a TNF homology domain (green, PRU01387). Post-translational modifications include disulfide bonds (pink) and N-linked glycosylation sites (violet). The 3D protein model illustrates the structural organization of these domains, highlighting the TNF homology domain as essential for receptor interactions.

*EDA*, a TNF superfamily cytokine, is associated with functions such as cytokine activity (GO:0005125), TNF receptor binding (GO:0005164), and death receptor binding (GO:0005123), mediating epithelial–mesenchymal signaling and NF-κB pathway activation essential for tooth development. Figure 5 depicts the genomic organization of EDA.

Based on previous findings, third molar agenesis was observed in 70% of individuals in the cohort, but in only 30% of patients with *EDA* gene mutations [13]. In a functional study performed using *EDA* deficient mice, it was shown that Eda deficiency affects Sonic hedgehog (Shh) signaling during incisor development [50], revealing that while early Shh expression remains largely unaffected, loss of Eda impairs enamel knot formation, leading to abnormal enamel organ morphogenesis and hypoplastic incisor formation.

**Figure 6:**
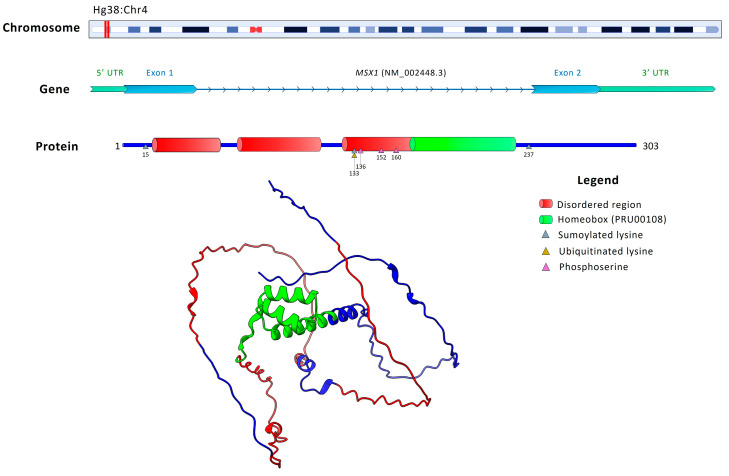
Chromosomal localization, gene structure, and protein domains of *MSX1* (NM_002448.3). The upper panel shows the localization of the *MSX1* gene on chromosome 4, with its position highlighted in red. The gene consists of only two exons. The encoded protein comprises 303 amino acids and includes disordered regions (red) and a homeobox domain (green, PRU00108), which is crucial for DNA binding and transcriptional regulation. Post-translational modifications are indicated, such as sumoylation, ubiquitination, and phosphorylation sites. The 3D protein model illustrates the structural organization of these domains, emphasizing the central role of the homeobox domain in regulating odontogenesis.

MSX1, a homeobox transcription factor, carries annotations for transcriptional repression (GO:0001227), odontogenesis (GO:0042476), and face morphogenesis (GO:0060325), highlighting its role in craniofacial patterning and tooth formation. Figure 6 illustrates the genomic organization of *MSX1*.

As reported in the IMPC database and supported by several functional studies [51,52], *MSX1* deficiency profoundly impacts craniofacial morphogenesis, particularly affecting lip and mandibular development. In *Msx1*-mutant mouse models, BMP signaling, which is essential for normal mandibular patterning and osteogenesis, was found to be significantly downregulated, underscoring the pivotal regulatory role of *MSX1* in craniofacial and dental tissue formation.

As subsequently described in Section 3.2, *PAX9*, *EDA* and *MSX1* collectively orchestrate transcriptional regulation and signaling processes fundamental to odontogenesis, explaining their frequent involvement in dental agenesis.

Additional specific genetic polymorphisms have been linked to TA. A cross-sectional study involving 273 individuals (86 with TA, 187 controls) examined variants in *BMP2* (rs235768, rs1005464), *BMP4* (rs17563), *RUNX2* (rs59983488, rs1200425), and *SMAD6* (rs3934908, rs2119261) [16]. Notably, the TT genotype of *BMP2* rs235768 was significantly associated with a higher risk of DA (*p* < 0.001), while the TT genotype of *SMAD6* rs3934908 was linked to third molar agenesis (*p* = 0.023). These findings support the role of specific single-nucleotide polymorphisms in isolated forms of TA. Nevertheless, the potential contribution of other genetic factors—such as copy number variants, chromosomal abnormalities, or intronic polymorphisms—cannot be excluded, as these may not be readily detected by standard next-generation sequencing techniques.

**Figure 7:**
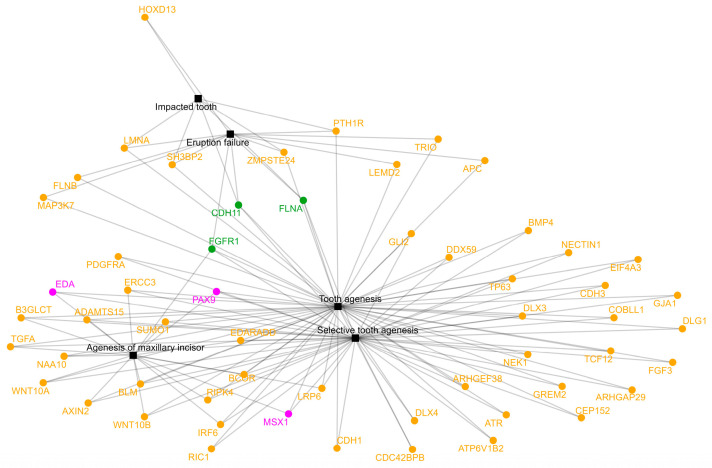
Network plot illustrating the gene–phenotype interaction for tooth agenesis and its related phenotypes. Only genes exhibiting at least two phenotypic associations are included. Phenotypes are represented by the black squares, while genes are shown by colored circles. Key genes with strong evidence for TA involvement (*EDA*, *PAX9*, and *MSX1*) are colored in magenta; *FGFR1*, *CDH11*, and *FLNA* genes showing the highest number of interactions (4) are colored in green; other associated genes are depicted in orange. The network emphasizes the polygenic nature of TA and illustrates the shared molecular basis among overlapping dental phenotypes. Data were retrieved from HPO database and elaborated using R studio version 3.4.3.

Another key consideration based on the gene–phenotype associations retrieved from HPO database and presented in Table 1, only three genes were found to be associated with 4 dental phenotypes, highlighting the variability in genotype–phenotype correlations in TA. Figure S1 presents a heatmap illustrating the association between genes and the different TA phenotypes, based on data extracted from the HPO database. Furthermore, Figure 7 represents a network plot with the association between the genes linked at least with 2 TA phenotypes retrieved from HPO database.

Specifically, *FGFR1* and *FLNA* were each linked to TA, selective tooth agenesis, agenesis of the maxillary incisor, and eruption failure. In contrast, *CDH11* was associated with TA, selective tooth agenesis, impacted tooth and eruption failure. These findings underscore the complexity of TA genetics and suggest that certain genes may contribute to broader craniofacial developmental pathways.

### 3.2. Signaling Pathways Associated with DA

Tooth agenesis is the result of disrupted odontogenesis, a highly coordinated process regulated by reciprocal epithelial–mesenchymal interactions [1,53,54]. Multiple signaling pathways are involved in initiation, morphogenesis, and eruption of teeth, and their dysregulation has been directly linked to congenital TA. The genetic basis of tooth agenesis is primarily linked to disruptions in genes regulating epithelial–mesenchymal interactions within the dental lamina [54]. Given the involvement of a wide array of signaling molecules—including transcription factors, growth factors, and receptors—a polygenic origin is likely [54]. Among the most studied pathways are the Wnt/β-catenin, *EDA*/*EDAR*/NF-κB, *BMP*/*TGF*-β, FGF, and SHH cascades, all of which orchestrate cell proliferation, differentiation, and tissue patterning in craniofacial development [53]. Mutations in genes encoding ligands, receptors, or downstream effectors of these pathways (e.g., *MSX1*, *PAX9*, *AXIN2*, *WNT10A*, *EDA*) disrupt enamel knot signaling and dental lamina formation, leading to hypodontia, oligodontia, anodontia or other dental malignancies such as tooth impaction [53,54,55]. Understanding these pathways provides crucial insights into the molecular mechanisms underlying tooth agenesis and offers potential avenues for targeted diagnosis and therapeutic approaches.

It is worth mentioning that these signaling pathways are also involved in broader biological processes such as tissue development and cancer, both of which are characterized by genetically driven cell proliferation. As a result, certain genes implicated in tooth agenesis, notably *AXIN2*, have been proposed as predictive cancer markers [54,56]. Indeed, several heterozygous AXIN2 variants have been identified in individuals with oligodontia who also developed lung, prostate, and colorectal cancers.

As documented, *LRP6* mutations were associated with TA [57]. A study in Chinese families with non-syndromic TA identified mutations in *AXIN2* and *LRP6*, linking both genes to the condition [58]. The same study also provided functional evidence that both suppression and excessive activation of the Wnt signaling pathway contribute to the pathogenesis of TA. Additionally, in a study of 14 patients with TA from eight families, five mutations in the *LRP6* gene were identified, including four missense variants (p.Glu72Lys, p.Lys82Asn, p.Tyr418His, and p.Ile773Val) and one nonsense variant (p.Arg32Ter) [59]. Beyond dental agenesis, *LRP6* mutations were associated with a broad spectrum of dental anomalies, such as mesiodens, tooth fusion, odontomas, microdontia, elongated roots, molars with unseparated roots, and taurodontism.

As widely documented, the Wnt signaling pathway plays a fundamental role in odontogenesis, regulating cell proliferation, differentiation, and the patterning of dental tissues [53]. Both canonical (β-catenin-dependent) and non-canonical Wnt signaling cascades are involved in tooth initiation and morphogenesis. Mutations in key Wnt pathway genes such as *WNT10A*, *WNT10B*, *AXIN2*, and *LRP6* have been strongly associated with dental agenesis, highlighting the pathway’s pivotal role in determining tooth number and position. As extensively documented, *MSX1* regulates Wnt signaling during early tooth development by suppressing the antagonists *DKK2* and *SFRP2*.

**Figure 8:**
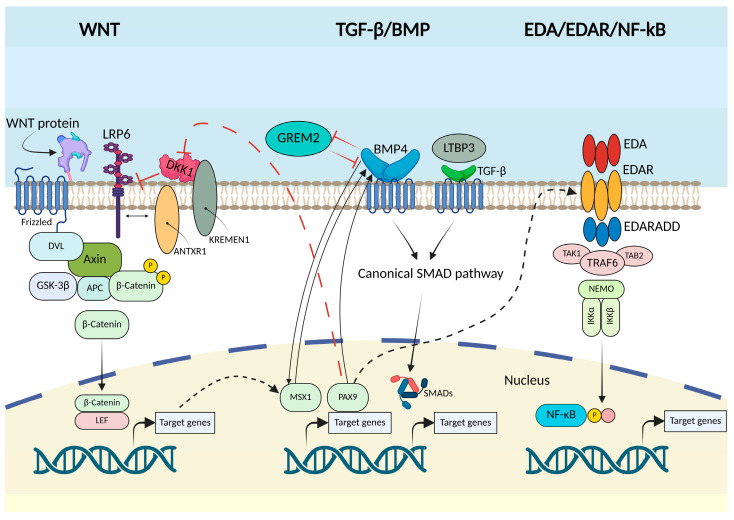
Molecular pathways implicated in tooth agenesis: Wnt, TGF-β/BMP, and EDA/EDAR/NF-κB. The figure illustrates three major signaling cascades involved in odontogenesis and disrupted in DA as result of mutations in the involved genes. In the Wnt pathway, WNT proteins (such as WNT10A and WNT10B) activate LRP6 and Frizzled receptors, stabilizing β-catenin and promoting transcription of odontogenic target genes, while regulators such as AXIN2 and antagonists like DKK1 and KREMEN1 modulate activity. The TGF-β/BMP pathway, mediated by ligands including BMP4 and TGF-β, signals through SMAD proteins to control transcription factors such as MSX1 and PAX9, with modulators like GREM2 and LTBP3 fine-tuning the process. The EDA/EDAR/NF-κB pathway is activated by EDA binding to EDAR and its adaptor proteins (EDARADD, TRAF6), leading to NF-κB nuclear translocation and regulation of tooth development genes. Together, these interconnected pathways orchestrate epithelial–mesenchymal signaling during tooth morphogenesis, and their disruption underlies both syndromic and non-syndromic forms of dental agenesis. This figure was generated using BioRender (https://BioRender.com) by Treccarichi, S. and adapted from a previous work [53].

As documented, homeobox genes play a central role in the signaling mechanisms that regulate odontogenesis, exerting specific actions at different stages of tooth development [60]. These transcription factors are key regulators of craniofacial morphogenesis through epithelial–mesenchymal interactions, and their dysregulation has been strongly linked to TA in both syndromic and non-syndromic forms. Among them, *MSX1*, *PAX9*, and *PITX2* are the most consistently associated with human TA, while DLX family members have also been implicated through experimental and animal model studies. Collectively, these genes act as critical determinants of tooth number, identity, and position. Figure 8 shows the molecular pathways Wnt, TGF-β/BMP, and EDA/EDAR/NF-κB implicated in TA.

**Figure 9:**
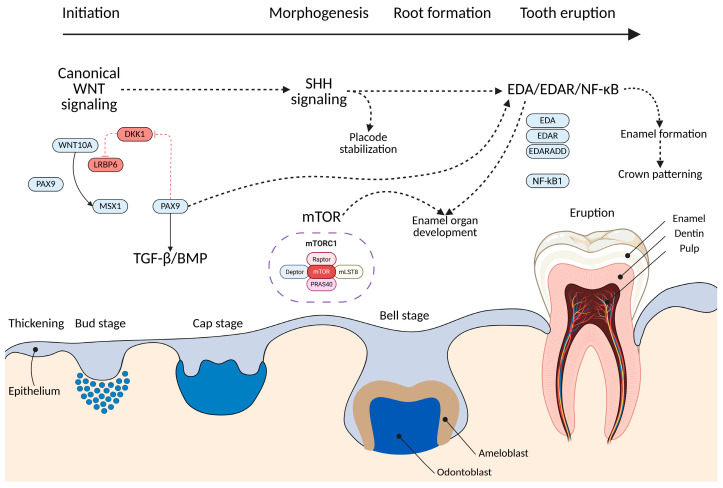
Mechanistic model of signaling pathways in odontogenesis. The figure displays the coordinated interaction of key signaling pathways—WNT, BMP/TGF-β, SHH, mTOR, and EDA/EDAR/NF-κB—across the stages of tooth development (initiation, morphogenesis, root formation, and eruption). Canonical WNT signaling via WNT10A, LRP6, MSX1, and PAX9 defines the odontogenic field and induces BMP/TGF-β activity. SHH signaling drives placode stabilization and cusp patterning, while mTORC1 regulates enamel organ growth, adhesion, and cytoskeletal organization. The EDA/EDAR/NF-κB pathway ensures epithelial integrity, governs enamel and crown morphogenesis, and contributes to root bifurcation through activity in Hertwig’s epithelial root sheath (HERS). The lower panel depicts the corresponding morphological progression—from epithelial thickening to bud, cap, and bell stages—culminating in enamel, dentin, and pulp formation. Collectively, these pathways act as an integrated network controlling tooth number, morphology, and integrity, whose disruption results in tooth agenesis.

As previously emphasized, tooth development is governed by a highly coordinated network of signaling pathways—WNT, BMP, EDA/NF-κB, SHH, and mTOR—that interact dynamically across epithelial–mesenchymal interfaces to regulate the sequential stages of odontogenesis [47,50,53]. Figure 9 graphically illustrates the dynamic interplay among the previously mentioned signaling pathways during the odontogenesis process, highlighting their coordinated roles in regulating tooth initiation, morphogenesis, root formation, and eruption.

During the initiation stage, canonical WNT signaling (mediated by WNT10A, LRP6, and AXIN2) establishes the dental placode by promoting epithelial proliferation and competence, while BMP signaling activates transcription factors such as MSX1 and PAX9 in the mesenchyme, defining the odontogenic field. The EDA/EDAR/NF-κB pathway refines placode spacing and maintains epithelial organization, ensuring proper enamel organ formation. As development advances, WNT-induced SHH expression orchestrates enamel knot formation and cusp morphogenesis, whereas BMP4 shapes crown architecture through precise modulation of morphogen gradients.

Concurrently, the mTOR signaling pathway plays a crucial metabolic and structural role, regulating cell adhesion, proliferation, and differentiation within the enamel organ. mTORC1 activity coordinates cytoskeletal organization and enamel matrix secretion, ensuring correct ameloblast function and tooth morphology [61]. In the root formation stage, EDA/EDAR signaling within Hertwig’s epithelial root sheath (HERS) governs epithelial proliferation and root bifurcation ensuring proper root architecture [62]. Collectively, these interconnected pathways form an integrated mechanistic framework in which balanced crosstalk between morphogenetic and metabolic signaling determines tooth number, shape, and structural integrity. Disruption of this network—via mutations in MSX1, PAX9, WNT10A, LRP6, EDA, or components of the mTOR complex—underlies the spectrum of phenotypic variability observed in TA.

According to Malacards database, Table 4 lists the top 10 pathways with the related genes associated with TA.

The most inferred pathway in MalaCards database was TNFs bind their physiological receptors, while the second one was mTOR pathway. The mTOR signaling pathway plays a crucial role in enamel organ development by regulating cell adhesion, proliferation, differentiation, and cytoskeletal dynamics [61]. Experimental evidence shows that mTOR deletion leads to enamel organ defects and tooth malformations, underscoring its importance in odontogenesis. In particular, the pathway acts primarily through the mTORC1 complex, and its dysregulation has been directly linked to the pathogenesis of tooth agenesis [61].

**Figure 10:**
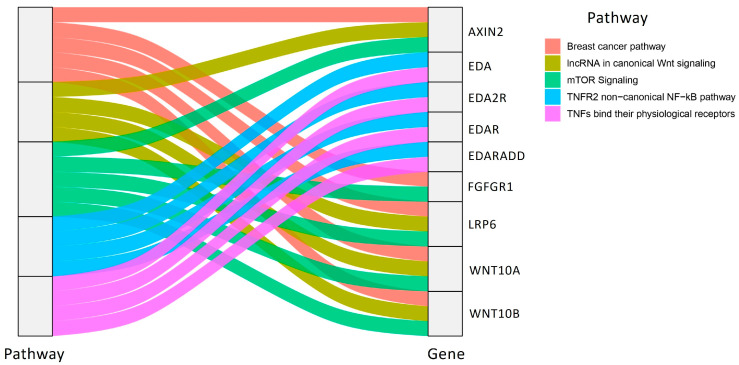
Sankey plot of pathway–gene associations in tooth agenesis. The diagram illustrates the relationships between major signaling pathways and the genes most frequently implicated in DA. Pathways include mTOR signaling, TNFR2 non-canonical NF-κB, TNFs binding their physiological receptors, lncRNA regulation of canonical Wnt signaling, and the breast cancer pathway. Key genes linked to these pathways are *AXIN2*, *EDA*, *EDA2R*, *EDAR*, *EDARADD*, *FGFR1*, *LRP6*, *WNT10A*, and *WNT10B*. The flow width represents the strength of association, highlighting how individual genes are shared across multiple pathways relevant to odontogenesis and TA pathogenesis. The gene-pathways associations were retrieved from MalaCards database, while the plot was generated using the package ggalluvial of R Studio software version 3.4.3.

The Sankey diagram illustrated in Figure 10 highlights the complex interplay between signaling pathways and genetic regulators involved in dental agenesis.

Notably, genes such as *AXIN2*, *LRP6*, and *WNT10A* appear across multiple pathways, underscoring their central role in Wnt-mediated odontogenesis. Similarly, components of the EDA/EDAR/NF-κB axis (*EDA*, *EDAR*, *EDARADD*) cluster within inflammatory and TNF-related pathways, reflecting their critical function in ectodermal development. The overlap of TA-associated genes with pathways commonly implicated in cancer. The EDA–EDAR–EDARADD signaling pathway plays a pivotal role in determining tooth number, crown morphology, and enamel formation. Evidence from studies on EDA-deficient mouse models has further demonstrated its crucial involvement in root development [62]. Specifically, disruption of this pathway alters Hertwig’s epithelial root sheath (HERS) morphology and proliferation, resulting in taurodontism due to impaired root bifurcation and abnormal HERS extension. Recent evidence has revealed intriguing overlaps between the molecular pathways implicated in breast cancer and those involved in tooth development, particularly within the Wnt, BMP, and PI3K–mTOR signaling networks. These pathways regulate key cellular processes such as proliferation, apoptosis, and differentiation, which are essential for both odontogenesis and tumorigenesis. Genes such as *FGFR1*, *WNT10A*, *AXIN2*, and *LRP6*—known contributors to tooth agenesis—also play established roles in breast cancer pathophysiology, suggesting shared mechanisms of aberrant signaling. In particular, AXIN2 variants, which alter Wnt signaling, have been associated with an increased predisposition to colorectal and breast cancer, reinforcing the concept that dysregulation of developmental pathways may predispose individuals to both dental anomalies and oncogenesis.

Several studies have highlighted the role of small RNAs, particularly microRNAs (miRNAs), ~20-nucleotide regulators that are crucial for tooth development [63]. By modulating the expression of genes within major epithelial–mesenchymal signaling pathways such as Wnt, BMP, and FGF, they control cell proliferation, differentiation, and apoptosis during odontogenesis. Dysregulation of specific miRNAs has been linked to tooth agenesis and impaction, as altered post-transcriptional control can disrupt enamel organ morphogenesis and odontogenic signaling cascades. A recent study curated 82 miRNAs associated with human odontogenesis and demonstrated, through functional enrichment analysis, that these miRNAs regulate pathways crucial for dental anomalies, including TGF-β and Wnt signaling as well as stem cell pluripotency [64]. Interestingly, several of the enriched pathways were also associated with tumorigenesis, such as small-cell lung and gastric cancer, underscoring the pleiotropic impact of miRNA-mediated regulation.

To investigate the regulatory roles of selected miRNAs in tooth development, it was performed a network analysis of their target genes, revealed several key hubs responsible for congenital DA [65]. Notably, hsa-miR-let-7a-3p targeted genes such as *CTNNB1*, *SOX9*, and *BMPR2*, while miR-15b-5p was linked to *FGF2*, *FGFR1/2*, and *AXIN2*. In addition, miR-200b-3p and hsa-miR-218-5p both converged on *ZEB2*, highlighting its potential role as a central hub in the odontogenic regulatory network [65].

## 4. Therapies and Future Perspectives

Future perspectives in the field of tooth agenesis focus on advancing the genetic understanding of both syndromic and non-syndromic forms, as well as developing innovative diagnostic tools. As recently documented, current artificial intelligence (AI) algorithms in dentistry focus on image-based diagnosis due to limited data and computational challenges in other tasks. While evidence-based dentistry remains the gold standard, machine learning serves as a supportive tool to enhance clinical decision-making [66]. Currently, virtual reality (VR)-based approaches are being applied in dentistry for diverse purposes, including oral hygiene education in individuals with dental agenesis or neurodevelopmental disorders and the management of dental anxiety [67,68,69]. One promising direction is the use of AI and machine learning algorithms to support pre-diagnostic and diagnostic assessments of missing teeth. In this context, the i-Dent project was developed as a pre-diagnostic tool designed to detect rare genetic oral and dental diseases through machine learning and deep learning techniques [70]. The system utilizes panoramic dental X-rays featuring varying patterns and severities of tooth agenesis as input. It is based on image segmentation models, such as Mask R–CNN and U-Net, which were extensively trained to enable automatic tooth detection on panoramic images. As documented, the i-Dent algorithm achieved an average accuracy of 72% and demonstrated the capability to identify three distinct patterns of missing teeth associated with pathogenic variants in *PAX9*, *WNT10A*, and *EDA* [70]. The model also showed potential for use in dental age estimation and early detection of rare genetic conditions, highlighting the crucial role of artificial intelligence in the future of precision dentistry.

Given their central role in regulating odontogenic signaling, miRNAs represent promising therapeutic targets for TA [65]. Modulating miRNA activity—either by restoring downregulated miRNAs with miRNA mimics or inhibiting overexpressed ones with antagomirs—could correct dysregulated pathways such as Wnt, BMP, or FGF signaling. We emphasize that further studies are needed to elucidate the functional relevance of miRNAs in TA and to assess their potential as therapeutic targets or diagnostic biomarkers. Notably, the detection of miRNAs in peripheral blood rather than dental tissues offers a minimally invasive approach, enabling earlier diagnosis, improved clinical management, and the development of personalized treatment strategies for patients with TA.

Such approaches have already shown potential in other developmental and regenerative contexts, suggesting that miRNA-based therapies could represent a future perspective, providing precision tools for preventing or directly treating TA. As outlined in a comprehensive review, studies in genetically modified mice suggest two main mechanisms for supernumerary tooth formation: rescue of arrested tooth germs (e.g., in *Runx2*/*Usag*-1 models) and the involvement of odontogenic epithelial stem cells [71]. Targeted molecular therapies, such as neutralizing antibodies, siRNAs, or small molecules, have been shown to stimulate tooth development or regeneration.

To date, whole-exome sequencing (WES) represents the most suitable clinical tool for detecting pathogenic variants associated with TA, allowing the identification of previously unreported mutations [72]. The integration of WES data with multi-omics approaches—including transcriptomics, proteomics, and epigenomics—could substantially enhance our understanding of the genetic architecture of TA, thereby expanding current knowledge and uncovering novel molecular mechanisms underlying this condition [73]. In fact, we emphasize that integrating dental data derived from radiographs with genetic variant data obtained through WES and in the near future, through long-read sequencing technologies—could represent a major advancement in understanding the etiology of dental agenesis. Future research should prioritize multi-omics studies, functional validation in animal and organoid models, and longitudinal clinical-genetic correlations to bridge molecular discoveries with patient-centered applications, ultimately advancing both diagnostic accuracy and regenerative treatment strategies in dental genetics. Improved accuracy in genetic analysis will enhance our understanding of the molecular and biochemical mechanisms underlying this condition. This, in turn, may pave the way for more precise approaches to the diagnosis, treatment, and clinical management of TA. From a therapeutic perspective, the management of tooth agenesis requires a multidisciplinary approach, often involving orthodontic, prosthetic, and surgical interventions tailored to the severity of tooth loss and patient age.

## 5. Conclusions

This review provides an updated synthesis of the genetic and molecular mechanisms underlying TA, highlighting the complex genetic architecture and the coordinated interplay of key signaling pathways that govern odontogenesis. Nevertheless, we acknowledge several limitations. We emphasize that our findings are largely based on secondary data from curated databases and previously published literature, which may introduce bias and limit the integration of novels, unpublished genetic evidence. Furthermore, the absence of a systematic methodology restricts quantitative assessment of gene–phenotype strength. Despite these limitations, we believe this review represents a valuable and forward-looking contribution for both researchers and clinicians, offering a solid foundation for advancing the understanding of the genetic mechanisms underlying tooth agenesis.

## Figures and Tables

**Table 1:** List of the phenotypes reported in the Human Phenotype Ontology (HPO) database (https://hpo.jax.org/) which includes tooth agenesis. Disease and gene column indicate the number of diseases and genes associated with each tooth agenesis phenotype.

ID	Name	Description	Disease (n)	Gene (n)
HP:0009804	Tooth agenesis	The absence of one or more teeth from the normal series by a failure to develop	351	361
HP:0001592	Selective tooth agenesis	Agenesis specifically affecting one of the classes incisor, premolar, or molar.	44	46
HP:0011079	Impacted tooth	A tooth that has not erupted because of local impediments	10	7
HP:0200160	Agenesis of maxillary incisor	Failure of development of maxillary incisor	8	19
HP:0000706	Eruption failure	A tooth which does not erupt within the teeth eruption timeline and after the loss of eruption potential.	30	17

**Table 2:** List of the syndromes caused by chromosomal abnormalities associated with tooth agenesis.

Name	Prevalence	Chromosomal Abnormality	Dental Abnormalities	Reference
Down syndrome	1 in 800–1200 live births	Trisomy 21	Hypodontia, delayed eruption, microdontia, taurodontism, abnormal crown morphology	[24,25]
Wolf–Hirschhorn syndrome	1 in 50,000 births	4p16.3 deletion	Tooth agenesis, delayed eruption, micrognathia	[32,33]
Turner syndrome	1 in 2000–2500 female births	Complete/partial monosomy X	Tooth agenesis (especially maxillary incisors, though not present in all individuals), supernumerary teeth, microdontia, enamel defects	[34]
Williams syndrome	1 in 7500–10,000 births	7q11.23 microdeletion	Hypodontia (50.9%), diastemas (72.5%), malocclusion, enamel defects, microdontia	[35,36]
DiGeorge syndrome	1 in 4000 births	22q11.2 deletion	Tooth agenesis, enamel hypoplasia, delayed eruption, cleft palate	[37,38]
Cri-du-chat syndrome	1 in 20,000–50,000 births	5p deletion	Tooth agenesis (not in all individuals), enamel hypoplasia, delayed eruption, dental malocclusion, microcephaly-related craniofacial anomalies	[42,43]

**Table 3:** Top 15 genes associated with dental agenesis according to the MalaCards database. The table reports for each gene the related pathways, the association score, and the corresponding Gene Ontology (GO) terms linked to the involved pathways.

Gene	Description	Score	Pathway
*MSX1*	Msh Homeobox 1	901.83	*GO:0030509*—bone morphogenetic protein (BMP) signaling pathway
*EDA*	Ectodysplasin A	814.5	*GO:0038061*—EDA-EDAR-EDARADD signaling pathway
*PAX9*	Paired Box 9	813.93	*GO:0030509*—bone morphogenetic protein (BMP) signaling pathway
*LRP6*	LDL Receptor Related Protein 6	800.69	*GO:0016055*—Wnt signaling pathway
*WNT10A*	Wnt Family Member 10A	778.59	*GO:0016055*—Wnt signaling pathway
*AXIN2*	Axin 2	427.18	*GO:0016055*—Wnt signaling pathway
*EDAR*	Ectodysplasin A Receptor	417.61	*GO:0038061*—EDA-EDAR-EDARADD signaling pathway
*ITPA*	Inosine Triphosphatase	400	*GO:0009124*—nucleoside monophosphate biosynthetic process
*RANBP2*	RAN Binding Protein 2	400	*GO:0005515*—protein binding
*EDARADD*	EDAR Associated Via Death Domain	383.89	*GO:0038061*—EDA-EDAR-EDARADD signaling pathway
*IRF6*	Interferon Regulatory Factor 6	375.6	*GO:0030509*—bone morphogenetic protein (BMP) signaling pathway
*WNT10B*	Wnt Family Member 10B	375.22	*GO:0016055*—Wnt signaling pathway
*GREM2*	Gremlin 2, DAN Family BMP Antagonist	374.85	*GO:0048063*—cytokine activity (BMP antagonist)
*TGFA*	Transforming Growth Factor Alpha	355.85	*GO:0005154*—epidermal growth factor receptor binding
*FGFR1*	Fibroblast Growth Factor Receptor 1	355.68	*GO:0004714*—transmembrane receptor protein tyrosine kinase activity

**Table 4:** List of the top 10 pathways associated with dental agenesis and annotated in the MalaCards database.

Pathway	Gene Set	Score	Involved Genes
TNFs bind their physiological receptors	4/28	6.99	*EDA*, *EDA2R*, *EDAR*, *EDARADD*
mTOR signaling	5/139	5.62	*AXIN2*, *FGFR1*, *LRP6*, *WNT10A*, *WNT10B*
Breast cancer pathway	5/154	5.4	*AXIN2*, *FGFR1*, *LRP6*, *WNT10A*, *WNT10B*
TNFR2 non-canonical NF-kB pathway	4/86	5	*EDA*, *EDA2R*, *EDAR*, *EDARADD*
lncRNA in canonical Wnt signaling and colorectal cancer	4/97	4.79	*AXIN2*, *LRP6*, *WNT10A*, *WNT10B*
Wnt signaling pathway and pluripotency	4/101	4.72	*AXIN2*, *LRP6*, *WNT10A*, *WNT10B*
Wnt signaling in kidney disease	3/37	4.56	*LRP6*, *WNT10A*, *WNT10B*
Wnt signaling pathways	4/114	4.51	*AXIN2*, *LRP6*, *WNT10A*, *WNT10B*
Embryonic stem cell pluripotency pathways	4/116	4.48	*FGFR1*, *LRP6*, *WNT10A*, *WNT10B*
Wnt pathway	4/134	4.24	*AXIN2*, *LRP6*, *WNT10A*, *WNT10B*

## Data Availability

No new data was created.

## Supplementary Materials

The following supporting information can be downloaded at https://www.mdpi.com/article/10.3390/ijms262110485/s1.

## Abbreviations

The following abbreviations are used in this manuscript:

DA	Dental agenesis
DS	Down syndrome
GWAS	Genome wide association study
HPO	Human Phenotype Ontology
ID	Intellectual disability
KS	Kabuki syndrome
TA	Tooth agenesis
TS	Turner Syndrome
WES	Whole exome sequencing
WHS	Wolf–Hirschhorn syndrome
WS	Williams syndrome
